# Macrobenthic fauna from an upwelling coastal area of Peru (Warm Temperate South-eastern Pacific province -Humboldtian ecoregion)

**DOI:** 10.3897/BDJ.6.e28937

**Published:** 2018-09-10

**Authors:** Vicente Tasso, Mustapha El Haddad, Carolina Assadi, Remy Canales, Luis Aguirre, Ximena Vélez-Zuazo

**Affiliations:** 1 Oceansnell, S.L. – Marine Environmental Consulting, Valencia, Spain Oceansnell, S.L. – Marine Environmental Consulting Valencia Spain; 2 Laboratorio de Biología y Sistemática de Invertebrados Marinos (LabSIM), Facultad de Ciencias Biológicas, Universidad Nacional Mayor de San Marcos, Lima, Peru Laboratorio de Biología y Sistemática de Invertebrados Marinos (LabSIM), Facultad de Ciencias Biológicas, Universidad Nacional Mayor de San Marcos Lima Peru; 3 Center for Conservation and Sustainability, Smithsonian Conservation Biology Institute, Washington, DC, United States of America Center for Conservation and Sustainability, Smithsonian Conservation Biology Institute Washington, DC United States of America

**Keywords:** Invertebrate assemblages, macrobenthos, neritic habitats, Humboldt current ecosystem, central coast, Peru, non-native species.

## Abstract

**Background:**

A total of 162 species and subspecies of marine macroinvertebrates were recorded in the submerged soft and hard substrates around the PERU LNG marine terminal and surrounding area, in the central coast of Peru, 167 km south of Lima, Peru. The collection of specimens was carried out from June 2011 to June 2015 as part of the research studies conducted by the Biodiversity Monitoring and Assessment Program (BMAP) around the marine terminal. The area is part of the Humboldt Current Large Marine Ecosystem, one of the most important upwelling systems in the world.

**New information:**

We identified specimens belonging to 83 families and seven phyla. The list was assembled from the taxonomic identifications made by the BMAP. We identified species and subspecies belonging to phyla Annelida, Arthropoda, Brachiopoda, Cnidaria, Echinodermata, Bryozoa and Mollusca. Phyla Annelida (60 spp.), Arthropoda (47 spp.)and Mollusca (45 spp.) exhibited the largest number of species.

## Introduction

Marine studies along the coastline of Peru are largely focused on species of economic importance (Tarazona et al. 2003). Species without economic importance or smaller size species, but of great importance for the maintenance and functioning of the marine ecosystem, remain poorly studied and understood. Marine invertebrates, particularly the coastal macrobenthic fauna, are known in Peru, but the information about them arises from studies focused on the effect of El Nino Southern Oscillation (ENSO) and largely limited to the fauna from deepest areas (Tarazona 1990, Tarazona et al. 1996, Arntz et al. 2006). This is understandable because, in deeper areas, the impact of ENSO on macrobenthic communities is more evident compared to shallow areas (Tarazona et al. 2003). This has resulted in a gap of information about the diversity and dynamics of the macrobenthos inhabiting shallow coastal areas (i.e. less than 15 metres). After the collapse of the anchovy fishery in the 1970s, research on marine biodiversity became more inclusive towards species without commercial importance (Arntz and Tarazona 1990). Preliminary research was focused on taxonomic groups already reported in lists and catalogues (Chirichigno and Vélez 1998, Del Solar et al. 1970, Mendez 1981, Alamo and Milla 1997, Paredes et al. 1988), but it eventually started to include less studied groups. Now, there is a greater record of research in biodiversity for different groups including decapods and stomatopods (Moscoso 2012), holothurians (Prieto 2010), asteroidea (Morales 2011), molluscs (Ramírez et al. 2003, Paredes et al. 2011, Cardoso et al. 2016), sponges (Azevedo et al. 2015), nudibranchs (Schrödl and Hooker 2014) and echinoids (Hooker et al. 2012). This remarkable increase of biodiversity information helps understanding the general macrobenthonic diversity and encourages the compilation of geographically-focused lists to improve our understanding about species range distribution and for monitoring temporal changes.

Here, we have assembled a taxonomic list of macrobenthic species present in the shallow coastal area near the international marine terminal of company PERU LNG (PLNG), in Pampa Melchorita, on the central coast of Peru, in the southeast Pacific. The area where the terminal is located is part of the Humboldt Current Large Marine Ecosystem (HCLME), an important upwelling system encompassing submerged habitats from the southern tip of Chile to northern Peru. The HCLME is considered amongst the most productive marine ecosystems in the world and knowledge about the diversity and natural processes characterising it are in great need, given its importance in global fisheries economy (Sherman 1991, Bakun and Weeks 2008). This taxonomic list focuses both on the species inhabiting the hard and soft bottoms at intertidal and subtidal levels in the area of direct influence of the marine terminal and control sites.

## Materials and methods

### Study area

The checklist of benthic macrofauna species was assembled using data collected by the Biodiversity Monitoring Assessment Program (BMAP). This programme is carried out in collaboration with the Smithsonian Conservation Biology Institute and PLNG. The area of study is the area of influence of PLNG marine terminal (13°15,15'S; 76°18,5'W), situated 167 km south of Lima, Peru. The submerged area is characterised by sediment flats with scattered patches of hard bottom and new artificial hard bottom created after establishment of an 800 m-long breakwater. Sampling was carried out close to the PLNG marine terminal and surroundings from June 2011 to June 2015, with a biennial periodicity. Samples were taken in three replicates both from soft and hard substrates (Suppl. material 1). Soft substrate samples were obtained from the resurgence and saturated zone from the intertidal (Salvat 1964) and from the subtidal at depths of 8, 10, 12 and 15 m (Fig. 1a). Samples collected from artificial hard substrates (breakwaters and piles) were obtained from the intertidal level (0 m) and subtidal levels (depths of 5 to 10 m) (Fig. 1b). To collect samples from intertidal soft substrate, an 18 cm-diameter benthos hand corer was used while, for the subtidal soft substrate, a 0.05 m^2^ Van Veen grab was used. Samples were sieved through a bag of 0.5 mm^2^ mesh size and the retained material was fixed with 4% formaldehyde in seawater. To facilitate later triage work and taxonomic analysis, the samples were stained with 1% rose bengal. To collect samples from hard substrate, a 25 cm x 25 cm quadrat was used and samples were obtained by clearing all specimens within the quadrat using a chisel and hammer.

### Identification of species and data analysis

The taxonomic identifications of collected specimens were made by the team of taxonomists from The Environment Management S.A.C (TEM). In this study, we only considered taxa identified at species or subspecies levels. Names of higher taxa as well as names of species and subspecies within them are listed alphabetically. For each of the species, we provide information about its original combination, the type of substrate (soft or hard), depth or bathymetric range, code of station where it was reported (with the name of transect and the depths in parentheses). We include remarks when necessary, particularly in the case of identified non-native species. Voucher specimens were deposited at the scientific collection of Laboratorio de Biología y Sistemática de Invertebrados Marinos (LabSIM) at Universidad Nacional Mayor de San Marcos (UNMSM).

We estimated species richness to test sampling effort using the non-parametric estimator that better fits our occurrence-data from multiple samples (Chao2, Chao 1984, Colwell and Coddington 1994), implemented in package Vegan in R (Oksanen et al. 2017). For this, we separated the species datasets sampling sites (i.e. soft-bottom and hard bottom, Fig. 1) and obtained rarefaction-based species accumulation curves.

## Data resources

**Data package title**: Macrobenthos_bmapperu

**Resource link**: http://ipt.pensoft.net/resource?r=macrobenthos_bmapperu&v=1.2

**Number of datasets**: 1

**Data set name**: Macrobenthos from upwelling coastal area of Peru

**Data format**: Darwin Core Archive (DwC-A)

## Checklists

### Annelida

#### Arenicolidae

#### Abarenicola affinis affinis

(Ashworth, 1902)

Arenicola assimilis affinis Ashworth, 1902

##### Notes

Types of substrate: soft bottom. Depth / bathymetric range: 0-15 m. Station code: BT1N(10, 12, 15); BT1S(12, 15); BT2N(0, 10, 12, 15); BT2S(12, 15); BT3N(15); BT3S(10, 15);BT4N(10, 15).

#### Capitellidae

#### Mediomastus branchiferus

Hartmann-Schröder, 1962

##### Notes

Types of substrate: hard and soft bottoms. Depth / bathymetric range: 0-15 m. Station code: BT1N (8, 10, 12); BT1S(8, 10, 12); BT2N(8, 10, 12, 15); BT2S(8, 12); BT3S(8); BT4N(8); D1(0, 10); D2(5, 10); D3(0, 5, 10); D4(0).

#### Maldanidae

#### Axiothella rubrocincta

(Johnson, 1901)

Clymenella rubrocincta Johnson, 1901

##### Notes

Types of substrate: hard bottom. Depth / bathymetric range: 5-10 m. Station code: D2(5, 10).

#### Cossuridae

#### Cossura chilensis

Hartmann-Schröder, 1965

##### Notes

**Figure 2.**
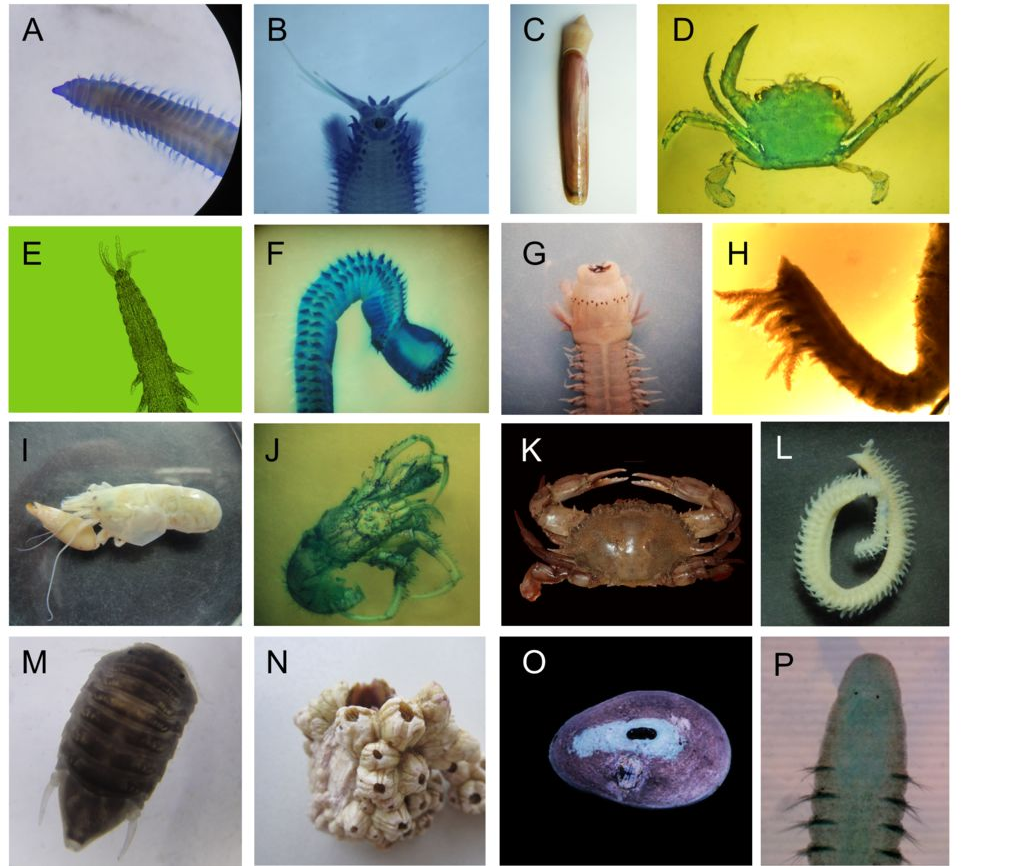
Species of invertebrates from central coast of Peru in the influence area of PERU LNG marine terminal. A. *Cossura chilensis*, B. *Diopatra chiliensis*, C. *Ensis macha*, D. *Cronius ruber*, E. *Goniadides falcigera*, F. *Nephtys impressa*, G. *Pseudonereis gallapagensis*, H. *Paraprionospio pinnata*, I. *Synalpheus spinifrons*, J. *Pagurus villosus*, K. *Arenaeus mexicanus*, L. *Scolelepis chilensis*, M. *Ancinus panamensis*, N. *Balanus laevis*, O. *Fissurella latimarginata*, P. *Protoariciella uncinata*.

Material examined: Fig. 2. Prostomium conical or almost triangular in shape, slightly longer than width; two peristomial segments, first incomplete peristomial segment, first setiger uniramous; branchiae on setigers 3. Types of substrate: soft bottom. Depth / bathymetric range: 10-15 m. Station code: BT1N(10, 12); BT1S(10, 12); BT2N(12, 15); BT2S(12); BT4N(15).

#### Dorvilleidae

#### Schistomeringos annulata

(Moore, 1906)

Stauronereis annulatus Moore, 1906

##### Notes

Types of substrate: hard bottom. Depth / bathymetric range: 0-10 m. Station code: D1(5); D2(5, 10); D3(0, 5, 10); D4(0, 5).

#### Lumbrineridae

#### Lumbrineris biuncinata

Hartmann-Schröder, 1960

##### Notes

Types of substrate: hard bottom. Depth / bathymetric range: 0-5 m. Station code: D1(5); D4(0, 5).

#### Lumbrineris lobata

Hartmann-Schröder, 1960

##### Notes

Types of substrate: hard and soft bottoms. Depth / bathymetric range: 0-10 m. Station code: BT2S(0); D1(0, 5, 10); D2(0, 5, 10); D3(0, 5, 10); D4(0, 10); D5(0).

#### Onuphidae

#### Diopatra chiliensis

Quatrefages, 1866

##### Notes

Material examined: Fig. 2. Prostomium rounded; Ceratophores of palps and antennae with 9-11 (some times 8-11) proximal rings and a longer distal ring. Anterior 5-6 pairs of parapodia modified, with 4–5 bidentate pseudocompound hooks and 1–2 upper simple chaetae, hooks with tiny spines on the shaft. Branchiae with up to 23 spiralled whorls, with long and thin filaments, starting from chaetiger 5. Types of substrate: soft bottom. Depth / bathymetric range: 8-15 m. Station code: BT1N(8, 10, 12, 15); BT1S(8, 10, 12, 15); BT2N(10, 12, 15); BT2S(8, 10, 12, 15); BT3N(15); BT3S(8, 10); BT4N(8, 10, 15).

#### Diopatra obliqua

Hartman, 1944

##### Notes

Types of substrate: hard and soft bottoms. Depth / bathymetric range: 8-15 m. Station code: BT1N(10); BT1S(8, 10, 12, 15); BT2N(8, 10); BT2S(8, 10, 12); BT3S(8, 10); MU(17).

#### Orbiniidae

#### Leitoscoloplos chilensis

(Hartmann-Schröder, 1965)

Haploscoloplos kerguelensis chilensis Hartmann-Schröder, 1965

##### Notes

Types of substrate: soft bottom. Depth / bathymetric range: 8-12 m. Station code: BT1N(8, 10); BT1S(10); BT2N(12); BT2S(10, 12); BT3N(10, 12); BT3S(8).

#### Leitoscoloplos kerguelensis

(McIntosh, 1885)

Scoloplos kerguelensis McIntosh, 1885

##### Notes

Types of substrate: soft bottom. Depth / bathymetric range: 8-12 m. Station code: BT1N(10); BT1S(8, 10); BT2S(12); BT4N(10).

#### Naineris brevicephala

Hartmann-Schröder, 1960

##### Notes

Types of substrate: hard bottom. Depth / bathymetric range: 0-10 m. Station code: D2(5); D3(0, 5, 10); D4(0, 10).

#### Protoariciella uncinata

Hartmann-Schröder, 1962

##### Notes

Material examined: Fig. 2. Prostomium rounded, as long as wide, a pair of eyes located in the posterior half of the prostomium; the division of the thorax and abdomen is not clear. The first two segments without chaetas. Cirrus dorsal short and digitiforms. Gills from segment 8. Types of substrate: hard and soft bottoms. Depth / bathymetric range: 0-10 m. Station code: BT3S(0); BT4N(8); D1(0, 5, 10); D2(0); D3(0, 5, 10); D4(0, 5).

#### Scoloplos rubra

(Webster, 1879)

Aricia rubra Webster, 1879

##### Notes

Types of substrate: soft bottom. Depth / bathymetric range: 0-12 m. Station code: BT1N(0, 8); BT1S(8, 12); BT2N(0).

#### Oweniidae

#### Owenia collaris

Hartman, 1955

##### Notes

Types of substrate: soft bottom. Depth / bathymetric range: 8-15 m. Station code: BT1N(8, 10, 12, 15); BT1S(8, 10, 12, 15); BT2N(8, 10, 12, 15); BT2S(8, 10, 12, 15); BT3N(8, 10, 15); BT3S(8, 10, 15); BT4N(8, 10, 15).

#### Glyceridae

#### Glycera americana

Leidy, 1855

##### Notes

Types of substrate: hard and soft bottoms. Depth / bathymetric range: 5-15 m. Station code: BT1N(0, 8, 10, 12); BT1S(8, 10, 12); BT2N(8, 10, 12, 15); BT2S(8, 10, 12); BT3N(8, 10, 12); BT3S(8); BT4N(8, 10); D3(5, 10)

#### Hemipodia californiensis

(Hartman, 1938)

Hemipodus californiensis Hartman, 1938

##### Notes

Types of substrate: hard and soft bottoms. Depth / bathymetric range: 0-12 m. Station code: BT1N(0, 8); BT1S(0); BT2N(0, 12); BT2S(0, 12); BT3S(0); BT4N(0); D2(0, 10).

#### Goniadidae

#### Goniada echinulata

Grube, 1870

##### Notes

Types of substrate: soft bottom. Depth / bathymetric range: 0-15 m. Station code: BT1N(8, 10, 12, 15); BT1S(0, 8, 10, 12); BT2N(8, 10, 12, 15); BT2S(0, 8, 10, 12, 15); BT3N(10); BT3S(8, 10); BT4N(8, 10).

#### Goniadides falcigera

Hartmann-Schröder, 1962

##### Notes

Material examined: Fig. 2. Prostomium with 8 rings, 4 antennae with constrictions resembling annulations. Proboscis with several different types of papillae, arranged in distinct longitudinal rows and best developed in median proboscidial part. Papillae long and unidentate, fang-shaped papillae with bent tip and broad base. Papillae slightly shorter, unidentate, fang-shaped papillae with slightly bent tip and smaller base. Papillae shorter, unidentate, more or less straight, conical papillae with slender base. Papillae small, stout conical to globular. Papillae slightly smaller, stout globular. Papillae slightly smaller, stout globular to rounded papillae. First segment usually without parapodium and chaetous. Black granulations in the parapodium and part of the body. Types of substrate: soft bottom. Depth / bathymetric range: 0-10 m. Station code: BT1N(0, 8, 10); BT1S(0); BT2N(0); BT2S(0, 10); BT3S(0); BT4N(0).

#### Hesionidae

#### Heteropodarke heteromorpha

Hartmann-Schröder, 1962

##### Notes

Types of substrate: soft bottom. Depth / bathymetric range: 8-10 m. Station code: BT2N(8, 10).

#### Oxydromus furcatus

(Hartmann-Schröder, 1962)

Podarke furcata Hartmann-Schröder, 1962

##### Notes

Types of substrate: hard bottom. Depth / bathymetric range: 0-10 m. Station code: D1(5); D2(5, 10); D3(0, 5, 10); D4(0, 5); D5(0).

#### Nephytyidae

#### Nephtys ferruginea

Hartman, 1940

##### Notes

Types of substrate: soft bottom. Depth / bathymetric range: 8-15 m. Station code: BT1N(10); BT1S(12, 15); BT2N(8, 10); BT3N(8); BT4N(8).

#### Nephtys impressa

Baird, 1873

##### Notes

Material examined: Fig. 2. The prostomium is approximately pentagonal in shape and broader than long. Long proboscis, with 22 rows of papillae, each row with 6 to 7 papillae, the anterior papillae are 2 to 3 times the size of the posterior ones. Interramal cirri first present on segment 4 and continuing through of body, distinctly recurved and heavily ciliated. The neuropodium carries a superior lobe present in the anterior and posterior segments. Types of substrate: soft bottom. Depth / bathymetric range: 0-15 m. Station code: BT1N(0, 8, 10, 12, 15); BT1S(8, 10, 12, 15); BT2N(8, 10, 12, 15); BT2S(8, 10, 12, 15); BT3N(8, 10, 12, 15); BT3S(0, 8, 10); BT4N(8, 10, 15).

#### Nereididae

#### Nereis callaona

(Grube, 1857)

Nereilepas callaona Grube, 1857

##### Notes

Types of substrate: hard and soft bottoms. Depth / bathymetric range: 0-15 m. Station code: BT1N(15); BT1S(10); BT4N(8); D1(0, 5, 10); D2(0, 5, 10); D3(0, 5, 10); D4(0, 5, 10); D5(0).

#### Platynereis australis

(Schmarda, 1861)

##### Notes

Types of substrate: hard bottom. Depth / bathymetric range: 0 m. Station code: D3(0).

#### Pseudonereis gallapagensis

Kinberg, 1865

##### Notes

Material examined: Fig. 2. Prostomium with entire anterior margin, wider than long. The dorsal part of the body presents a greenish-brown colour, including the prostomium and palps. One apodous anterior segment, greater than length of chaetiger 1. Tentacular cirri with distinct cirrophores, longest tentacular cirri extend back to chaetiger 3–4. The paragnath distribution: area I: 1 conical paragnath; area II: about 17-20 bar paragnaths in four rows; area III: Numerous paragnaths distributed in 4 rows; area IV: about 40–50 bar paragnaths in 4 rows, around 15 cones towards jaws and 2–4 bars next to the jaws, area V: 1 conical paragnath; area VI: 1 large triangular shield-shaped bar present; area VII and VIII: about 18–20 in two rows, anterior row with cones, posterior with bars, forming a single band of paragnaths. First two parapodia uniramous, all others biramous. Long dorsal cirrus, located at the distal end of the dorsal lobe of the notopodium from the posterior third of the body. Types of substrate: hard bottom. Depth / bathymetric range: 0-10 m. Station code: D1(0, 5, 10); D2(0); D3(0, 10); D4(0, 5, 10); D5(0).

#### Phyllodocidae

#### Phyllodoce parvula

Gravier, 1907

##### Notes

Types of substrate: hard bottom. Depth / bathymetric range: 0-10 m. Station code: D3(0, 5, 10); D4(0).

#### Protomystides confusa

Hartmann-Schröder, 1962

##### Notes

Types of substrate: hard bottom. Depth / bathymetric range: 0-10 m. Station code: D1(10); D2(0, 10); D3(5, 10); D4(0).

#### Protomystides lanceolata

Hartmann-Schröder, 1962

##### Notes

Types of substrate: hard bottom. Depth / bathymetric range: 0-10 m. Station code: D1(5); D2(0, 5, 10); D3(0, 5, 10); D4(0, 5); D5(5).

#### Steggoa negra

Hartmann-Schröder, 1962

##### Notes

Types of substrate: hard and soft bottoms. Depth / bathymetric range: 0-12 m. Station code: BT1N(4); BT1S(8, 10); BT2N(8, 10); BT2S(8, 10, 12); BT3S(8, 10); BT4N(8); D1(0); D2(0); D3(0, 5, 10).

#### Steggoa peruana

Hartmann-Schröder, 1960

##### Notes

Types of substrate: hard bottom. Depth / bathymetric range: 0-10 m. Station code: D1(5, 10); D2(0, 5, 10); D3(0, 5, 10); D4(0, 5); D5(0).

#### Pilargidae

#### Hermundura fauveli

(Berkeley & Berkeley, 1941)

Loandalia fauveli Berkeley & Berkeley, 1941

##### Notes

Types of substrate: hard and soft bottoms. Depth / bathymetric range: 0-15 m. Station code: BT1N(8, 10, 12,15); BT1S(10, 12, 15); BT2N(10, 12, 15); BT2S(12, 15); BT3N(12); BT3S(0, 10, 15); BT4N(10, 15); D3(0).

#### Sigambra bassi

(Hartman, 1945)

Ancistrosyllis bassi Hartman, 1945

##### Notes

Types of substrate: soft bottom. Depth / bathymetric range: 10 m. Station code: BT1N(10); BT1S(10); BT4N(10).

#### Polynoidae

#### Halosydna fuscomarmorata

(Grube, 1876)

Polynoe fuscomarmorata Grube, 1876

##### Notes

Types of substrate: hard bottom. Depth / bathymetric range: 0-10 m. Station code: D1(0); D2(5, 10); D3(0, 5, 10); D4(0); D5(0).

#### Halosydna johnsoni

(Darboux, 1899)

Lepidonotus johnsoni Darboux, 1899

##### Notes

Types of substrate: hard bottom. Depth / bathymetric range: 0-10 m. Station code: D1(0, 5, 10); D2(0, 5, 10); D3(0, 5, 10); D4(0, 5, 10); D5(0).

#### Halosydna parva

Kinberg, 1856

##### Notes

Types of substrate: hard bottom. Depth / bathymetric range: 0-10 m. Station code: D3(0, 5, 10); D4(0).

#### Harmothoe hirsuta

Johnson, 1897

##### Notes

Types of substrate: hard bottom. Depth / bathymetric range: 10 m. Station code: D3(10).

#### Lepidonotus crosslandi peruana

Hartmann-Schröder, 1962

##### Notes

Types of substrate: hard bottom. Depth / bathymetric range: 0-10 m. Station code: D1(0, 5, 10); D2(10); D3(0, 5, 10); D4(0).

#### Sigalionidae

#### Pholoides asperus

(Johnson, 1897)

Peisidice aspera Johnson, 1897

##### Notes

Types of substrate: hard bottom. Depth / bathymetric range: 10 m. Station code: D2(10).

#### Pisione koepkei

Siewing, 1955

##### Notes

Types of substrate: soft bottom. Depth / bathymetric range: 0 m. Station code: BT1N(0); BT1S(0); BT2N(0); BT2S(0); BT3S(0); BT4N(0).

#### Sthenelais helenae

Kinberg, 1856

##### Notes

Types of substrate: soft bottom. Depth / bathymetric range: 10-15 m. Station code: BT1S(10); BT4N(15).

#### Syllidae

#### Eusyllis liniata

(Hartmann-Schröder, 1962)

Odontosyllis liniata Hartmann-Schröder, 1962

##### Notes

Types of substrate: hard bottom. Depth / bathymetric range: 0-10 m. Station code: D1(5, 10); D2(0, 10); D3(0, 5, 10); D4(0).

#### Myrianida paredesi

Aguirre, San Martín & Álvarez-campos, 2015

##### Notes

Types of substrate: hard bottom. Depth / bathymetric range: 10 m. Station code: D1(10); D2(10); D3(10). Remarks: This species was described recently from the study area (Aguirre et al. 2015)

#### Proceraea micropedata

(Hartmann-Schröder, 1962)

Odontosyllis micropedata Hartmann-Schröder, 1962

##### Notes

Types of substrate: hard bottom. Depth / bathymetric range: 0-10 m. Station code: D1(10); D2(5, 10); D3(0, 5, 10); D4(10).

#### Syllis magdalena

(Ehlers, 1901)

Syllis prolixa Ehlers, 1901

##### Notes

Types of substrate: hard bottom. Depth / bathymetric range: 0-10 m. Station code: D1(10); D2(10); D3(0, 10); D4(0).

#### Sabellidae

#### Paradialychone paracincta

(Hartmann-Schröder, 1962)

Chone paracincta Hartmann-Schröder, 1962

##### Notes

Types of substrate: hard and soft bottoms. Depth / bathymetric range: 8- 10 m. Station code:BT1N(8); BT2N(10); D3(10).

#### Parasabella leucaspis

(Kinberg, 1867)

Demonax leucaspis Kinberg, 1867

##### Notes

Types of substrate: hard bottom. Depth / bathymetric range: 10 m. Station code: D1(10); D2(10); D3(10).

#### Spionidae

#### Boccardia polybranchia

(Haswell, 1885)

Polydora polybranchia Haswell, 1885

##### Notes

Types of substrate: hard bottom. Depth / bathymetric range: 0-10 m. Station code: D1(0, 10); D2(0, 5, 10); D3(0, 5, 10); D4(0, 10).

#### Carazziella carrascoi

Blake, 1979

##### Notes

Types of substrate: hard bottom. Depth / bathymetric range: 0 m. Station code: D3(0); D4(0).

#### Dipolydora socialis

(Schmarda, 1861)

Leucodore sociales Schmarda, 1861

##### Notes

Types of substrate: hard bottom. Depth / bathymetric range: 5-10 m. Station code: D2(5, 10); D3(5, 10).

#### Paraprionospio pinnata

(Ehlers, 1901)

Prionospio (Paraprionospio) pinnata Ehlers, 1901

##### Notes

Material examined: Fig. 2. Prostomium fusiform with rounded anterior border. Peristomium with projections that wrap dorsolaterally to the prostomium. Palp with basal sheath. Three pairs of branchiae on setigers 1–3. Each carries numerous lamellae; the lamellae of the first pair of branchiae are the largest. Notopodial postsetal lamellae elongate subtriangular on setigers 1–3, becoming low rounded posteriorly to about setiger 11 reducing in size. Anterior neuropodial postsetal lamellae ovate, distally pointed, becoming low rounded from setiger 4; lamellae reduced to a low ridge from setiger 9. Neuropodial hooded hooks, attaining 10–13 per fascicle. Neuropodial and notopodial hooded hooks with 3–4 pairs of apical teeth above main fang. Types of substrate: soft bottom. Depth / bathymetric range: 10-12 m. Station code: BT1S(10, 12).

#### Polydora aggregata

Blake, 1969

##### Notes

Types of substrate: hard and soft bottoms. Depth / bathymetric range: 0-12 m. Station code: BT1N(10); BT1S(8, 10, 12); D1(5); D2(0); D3(0, 5, 10); D4(0).

#### Polydora websteri

Hartman in Loosanoff & Engle, 1943

##### Notes

Types of substrate: hard bottom. Depth / bathymetric range: 0-10 m. Station code: D1(0, 5, 10); D2(0, 5, 10) D3(0, 5, 10); D4(0, 5). Remarks: considered as non-native species, with North American Pacific coast being its native distribution area (Cinar 2012). Considered as an invasive species in Hawaii Islands (Cinar 2012), Venezuela and Australia (Díaz and Liñero-Arana 2009), which has caused extensive damage to oysters.

#### Prionospio peruana

Hartmann-Schröder, 1962

##### Notes

Types of substrate: soft bottom. Depth / bathymetric range: 8-15 m. Station code: BT1N(8, 10, 12, 15); BT1S(8, 10, 12, 15); BT2N(8, 10, 12, 15); BT2S(8, 10, 12, 15); BT3N(10, 12, 15); BT3S(8, 10); BT4N(8, 10, 15).

#### Rhynchospio glutaea

(Ehlers, 1897)

Scolecolepis glutaea Ehlers, 1897

##### Notes

Types of substrate: hard and soft bottoms. Depth / bathymetric range: 0-10 m. Station code: BT1N(10); BT1S(10); BT2N(10); BT2S(10); D3(5); D4(0).

#### Scolelepis chilensis

(Hartmann-Schröder, 1962)

Nerine cirratulus chilensis Hartmann-Schröder, 1962

##### Notes

Material examined: Fig. 2. Prostomium elongated and distally pointed, continuing posteriorly as caruncle to end of setiger 1. Peristomium well developed, forming lateral wings that sometimes cover half of the prostomium. Setiger 1 reduced. Branchiae from setiger 7, fused with the dorsal lamella, leaving free only the tips of both; from the setigero 22-25, the fusion only covers half of the branchiae and lamella. Hooded hooks begining in neuropodia from setigers 25. Types of substrate: soft bottom. Depth / bathymetric range: 0-15 m. Station code: BT1N(0, 8); BT1S(8, 10); BT2N(8, 10, 12); BT2S(8,10, 12); BT3N(8, 10, 15); BT3S(8, 10,15); BT4N(8, 10).

#### Scolelepis squamata

(O.F. Muller, 1806)

Lumbricus squamatus O.F. Müller, 1806

##### Notes

Types of substrate: soft bottom. Depth / bathymetric range: 8-112 m. Station code: BT1N(10); BT1S(8, 10); BT2N(10); BT2S(8,10, 12); BT3S(8); BT4N(8).

#### Pectinariidae

#### Pectinaria chilensis

Nilsson, 1928

##### Notes

Types of substrate: hard and soft bottom. Depth / bathymetric range: 10-15 m. Station code: BT1N(12); BT1S(12, 15); BT2N(15); BT2S(15); BT3S(10, 15); D1(10).

#### Sabellariidae

#### Phragmatopoma virgini

Kinberg, 1866

##### Notes

Types of substrate: hard and soft bottoms. Depth / bathymetric range: 0-10 m. Station code: BT1N(10); D1(5, 10); D2(0, 5, 10); D3(0, 5, 10); D4(0, 5, 10).

#### Tereballidae

#### Pista mirabilis

McIntosh, 1885

##### Notes

Types of substrate: hard bottom. Depth / bathymetric range: 10 m. Station code: D3(10).

### Arthropoda

#### Caprellidae

#### Caprella scaura

Templeton, 1836

##### Notes

Types of substrate: hard and soft bottom. Depth / bathymetric range: 5-12 m. Station code: BT1N(12); BT2N(10); BT1S(12); D2(5). Remarks: considered as non-native species, being native to the western Indian (Martínez and Adarraga 2008).

#### Corophiidae

#### Monocorophium acherusicum

(Costa, 1853)

Corophium acherusicum Costa, 1853

##### Notes

Types of substrate: hard bottom. Depth / bathymetric range: 0-10 m. Station code: D1(5); D2(5, 10); D3(0, 5, 10). Remarks: considered as non-native species in Chile (Pérez-Schultheiss 2009). Its native area is not clear, probably Oriental Atlantic. Introduced probably by shipping as fouling.

#### Monocorophium insidiosum

(Crawford, 1937)

Corophium insidiosum Crawford, 1937

##### Notes

Types of substrate: hard and soft bottom. Depth / bathymetric range: 0-15 m. Station code: BT1N(15); BT2N(15); BT3S(15), D1(0, 5, 10); D2(5, 10); D3(0, 5, 10); D4(0, 10). Remarks: considered as non-native species in Chile (Pérez-Schultheiss 2009). After Fofonoff et al. 2003, it is native from Oriental Atlantic. Probably introduced by shipping as fouling.

#### Ischyroceridae

#### Ericthonius punctatus

(Bate, 1857)

Podocerus punctatus Bate, 1857

##### Notes

Types of substrate: hard bottom. Depth / bathymetric range: 0-10 m. Station code: D1 (0, 5, 10); D2 (5, 10); D3(0, 5, 10); D4(0, 10).

#### Maeridae

#### Elasmopus rapax

Costa, 1853

##### Notes

Types of substrate: hard and soft bottoms. Depth / bathymetric range: 0-15 m. Station code: BT1S(8, 15); BT2S(10), BT3N(15); BT3S(8); D1(0, 5, 10); D2(0, 5, 10); D3(0,5,10); D4(0, 5); D5(0). Remarks: Considered as non-native species from the Pacific (Pérez-Schultheiss 2009, Hughes and Lowry 2010). After Hughes and Lowry 2010, its origin can be Mediterranean, Red Sea or the Indian Ocean. Probably introduced by shipping as fouling.

#### Stenothoidae

#### Stenothoe valida

Dana, 1852

##### Notes

Types of substrate: Hard and soft bottoms. Depth / bathymetric range: 0-15 m. Station code: BT1N(15); D2(5); D3(0, 5, 10).

#### Aethridae

#### Hepatus lineatus

Rathbun, 1898

##### Notes

Types of substrate: soft bottom. Depth / bathymetric range: 10-12 m. Station code: BT1S(10); BT2S(12). Remarks: This species has a North American distribution (Hendrickx 1995) and for this reason can be considered as non-native species.

#### Albuneidae

#### Lepidopa deamae

Benedict, 1903

##### Notes

Types of substrate: soft bottom. Depth / bathymetric range: 0-8 m. Station code: BT1S(0); BT2S(8).

#### Alpheidae

#### Alpheus chilensis

Lenz, 1902

##### Notes

Types of substrate: hard bottom. Depth / bathymetric range: 5 m. Station code: D3(5).

#### Alpheus inca

Wicksten & Méndez, 1981

##### Notes

Types of substrate: hard bottom. Depth / bathymetric range: 5-10 m. Station code: D3(5, 10).

#### Synalpheus spinifrons

(Milne Edwards, 1837)

Alpheus spinifrons Milne Edwards, 1837 [in Milne Edwards, 1834-1840]:

##### Notes

Material examined: Fig. 2. Smooth caparace whose frontal border extends on a rostriform keel above the eyes that are protected by the border of the caparace. Chelas whose dactyl has a molariform tubercle and is modified in the form of a trigger. Periopods without epipodites. Types of substrate: hard bottom. Depth / bathymetric range: 0-10 m. Station code: D1 (5); D3(0, 5, 10).

#### Cancridae

#### Romaleon polyodon

(Latreille, 1802)

Cancer polyodon Poeppig, 1836

##### Notes

Types of substrate: hard bottom. Depth / bathymetric range: 0 m. Station code: D3(0).

#### Epialtidae

#### Acanthonyx petiverii

Milne Edwards, 1834

##### Notes

Types of substrate: hard bottom. Depth / bathymetric range: 0 m. Station code: D3(0).

#### Epialtidae

#### Pachygrapsus transversus

(Gibbes, 1850)

Grapsus transversus Gibbes, 1850

##### Notes

Types of substrate: hard bottom. Depth / bathymetric range: 0 m. Station code: D1(0); D3(0); D5(0).

#### Hippidae

#### Emerita analoga

(Stimpson, 1857)

Hippa analoga Stimpson, 1857

##### Notes

Types of substrate: soft bottom. Depth / bathymetric range: 0-8 m. Station code: BT1N(0); BT1S(0); BT2N(0); BT2S(0, 8); BT3S(0); BT4N(0).

#### Paguridae

#### Pagurus perlatus

Milne Edwards, 1848

##### Notes

Types of substrate: soft bottom. Depth / bathymetric range: 8-10 m. Station code: BT1S(8); BT2N(8); BT2S(10); BT3S(8).

#### Pagurus villosus

Nicolet, 1849

##### Notes

Material examined: Fig. 2. Ocular acicles with single terminal spine. Antennal flagellum with long, evenly paired setae. Carpus of left cheliped with row of spines on both dorsomesial and dorsolateral margins; posterolateral telsonal plates composed of individual spinules or denticules. Types of substrate: soft bottom. Depth / bathymetric range: 8 m. Station code: BT3S(8).

#### Panopeidae

#### Eurypanopeus transversus

(Stimpson, 1860)

Panopeus transversus Stimpson, 1860

##### Notes

Types of substrate: hard bottom. Depth / bathymetric range: 0-10 m. Station code: D1(5); D2(0, 10), D3(0, 5, 10).

#### Pilumnoididae

#### Pilumnoides perlatus

(Poeppig, 1836)

Hepatus perlatus Poeppig, 1836

##### Notes

Types of substrate: hard bottom. Depth / bathymetric range: 0-10 m. Station code: D1(0, 5, 10); D2(0, 5, 10); D3(0, 5, 10); D4(0, 5, 10); D5(0).

#### Pinnotheridae

#### Calyptraeotheres politus

(Smith, 1870)

Ostracotheres politus Smith, 1870

##### Notes

Types of substrate: hard bottom. Depth / bathymetric range: 0-10 m. Station code: D1(10); D2(10); D3(0, 10).

#### Pinnixa valdiviensis

Rathbun, 1907

##### Notes

Types of substrate: soft bottom. Depth / bathymetric range: 8-15 m. Station code: BT1N(10); BT1S(15); BT2N(8, 15).

#### Platyxanthidae

#### Platyxanthus orbignyi

(Milne Edwards & Lucas, 1843)

Xantho orbignyi Milne Edwards & Lucas, 1843

##### Notes

Types of substrate: hard bottom. Depth / bathymetric range: 0-10 m. Station code: D2(10); D3(0, 5, 10).

#### Porcellanidae

#### Allopetrolisthes angulosus

(Guérin, 1835)

Porcellana angulosa Guérin, 1835

##### Notes

Types of substrate: hard bottom. Depth / bathymetric range: 0-5 m. Station code: D1(5); D3(0).

#### Allopetrolisthes punctatus

(Guérin, 1835)

Porcellana punctata Guérin, 1835

##### Notes

Types of substrate: hard bottom. Depth / bathymetric range: 0 m. Station code: D3(0); D5(0).

#### Allopetrolisthes spinifrons

(Milne Edwards, 1837)

Porcellana spinifrons Milne Edwards, 1837

##### Notes

Types of substrate: hard bottom. Depth / bathymetric range: 5 m. Station code: D4(5).

#### Liopetrolisthes mitra

(Dana, 1852)

Porcellana mitra Dana, 1852

##### Notes

Types of substrate: hard bottom. Depth / bathymetric range: 0-10 m. Station code: D1(0, 5, 10); D2(5); D3(0, 5, 10); D4(0, 5, 10); D5(0, 5).

#### Pachycheles crinimanus

Haig, 1960

##### Notes

Types of substrate: hard bottom. Depth / bathymetric range: 0-10 m. Station code: D1(0, 5); D2(0); D3(0, 5, 10); D4(0); D5(5).

#### Pachycheles grossimanus

(Guérin, 1835)

Porcellana grossimana Guérin, 1835

##### Notes

Types of substrate: hard bottom. Depth / bathymetric range: 10 m. Station code: D3(10).

#### Petrolisthes armatus

Gibbes, 1850

##### Notes

Types of substrate: hard bottom. Depth / bathymetric range: 5 m. Station code: D1(5); D2(5)

#### Petrolisthes desmarestii

(Guérin, 1835)

Porcellana desmarestii Guérin, 1835

##### Notes

Types of substrate: hard bottom. Depth / bathymetric range: 10 m. Station code: D3(10).

#### Petrolisthes granulosus

(Guérin, 1835)

Porcellana granulosa Guérin, 1835

##### Notes

Types of substrate: hard bottom. Depth / bathymetric range: 0-5 m. Station code: D2(5); D3(0, 5).

#### Petrolisthes laevigatus

(Guérin, 1835)

Porcellana laevigata Guérin, 1835

##### Notes

Types of substrate: hard bottom. Depth / bathymetric range: 0-5 m. Station code: D3(0, 5).

#### Portunidae

#### Arenaeus mexicanus

(Gerstaecker, 1856)

Euctenota mexicanus Gerstaecker, 1856

##### Notes

Material examined: Fig. 2. Caparace with 9 equal or subequal antero-latereales teeth. Eye peduncles much shorter than a third of the width of the caparace; deep supraorbital fissure, wide and "V" shaped. External surface of the cheliped palm with 1 or 2 well-marked spines. Types of substrate: soft bottom. Depth / bathymetric range: 8 m. Station code: D1(10); BT1S3(8).

#### Cronius ruber

(Lamarck, 1818)

Portunus ruber Lamarck, 1818

##### Notes

Material examined: Fig. 2. It differs from the other blue crabs with 9 antero-lateral spines in the their long and short alternations and the presence on the palm-surface with sharp and black tips. Types of substrate: hard bottom. Depth / bathymetric range: 5-10 m. Station code: D1(10); D3(5).

#### Varunidae

#### Pseudograpsus setosus

(Fabricius, 1798)

Cancer setosus Fabricius, 1798

##### Notes

Types of substrate: hard bottom. Depth / bathymetric range: 5-10 m. Station code: D1(10); D2(10); D3(5).

#### Xanthidae

#### Gaudichaudia gaudichaudii

(Milne Edwards, 1834)

Xantho gaudichaudii Milne Edwards, 1834

##### Notes

Types of substrate: hard bottom. Depth / bathymetric range: 0-10 m. Station code: D1(5); D2(5); D3(0, 10).

#### Gaudichaudia tridentatus

(Lenz, 1902)

Leptodius tridentatus Lenz, 1902

##### Notes

Types of substrate: hard bottom. Depth / bathymetric range: 0-10 m. Station code: D2(5); D3(0, 5, 10).

#### Ancinidae

#### Ancinus brasiliensis

Lemos de Castro, 1959

##### Notes

Types of substrate: soft bottom. Depth / bathymetric range: 10 m. Station code: BT1N(10). Remarks: It is a species from Western Atlantic (Glynn and Glynn 1974) probably introduced by maritime traffic.

#### Ancinus panamensis

Glynn & Glynn, 1974

##### Notes

Material examined: Fig. 2. Uropods uniramous; pereopod I subchelate; pereopod II subchelate in male only; cephalon medially fused to first pereonite. Pleotelson with transverse depression near posterior apex; lateral margin of rostrum straight, not anteriorly expanded; male pereopod II dactyl closing midway on propus. Types of substrate: soft bottom. Depth / bathymetric range: 8 m. Station code: BT1S(8); BT2N(8, 10); BT2S(8); BT3N(8).

#### Cirolanidae

#### Excirolana braziliensis

Richardson, 1912

##### Notes

Types of substrate: soft bottom. Depth / bathymetric range: 0-10 m. Station code: BT1N(0); BT1S(0); BT2N(0); BT2S(0, 10); BT3S(0); BT4N(0).

#### Idoteidae

#### Edotia transversa

Menzies, 1962

##### Notes

Types of substrate: soft bottom. Depth / bathymetric range: 10-12 m. Station code: BT1N(10, 12).

#### Sphaeromatidae

#### Paradella bakeri

(Menzies, 1962)

Dynamenopsis bakeri Menzies, 1962

##### Notes

Types of substrate: hard bottom. Depth / bathymetric range: 0-10 m. Station code: D1(0, 5); D2(0); D3(0, 5); D4(0, 10).

#### Balanidae

#### Austromegabalanus psittacus

(Molina, 1782)

Lepas psittacus Molina, 1782

##### Notes

Types of substrate: hard bottom. Depth / bathymetric range: 0-10 m. Station code: D1(0, 5, 10); D2(0, 5, 10); D3(0, 5, 10); D4(0, 10); D5(0).

#### Balanus laevis

Bruguière, 1789

##### Notes

Material examined: Fig. 2. The plates form a conical structure, rounded border and smooth margins. There are six narrow spokes on the surface corresponding to the sutures of the tables: four anterior spokes (corresponding to their joints of the Carina-carinolateral and carino-lateral plates) and two posterior radii (corresponding to their joints of the Rostral-lateral plates). Types of substrate: hard and soft bottoms. Depth / bathymetric range: 0-10 m. Station code: BT1S(8); BT2S(8); D1(0, 5, 10); D2(0, 5, 10); D3(0, 5, 10); D4(0, 5, 10); D5(0, 5).

#### Megabalanus tintinnabulum

(Linnaeus, 1758)

Balanus tintinnabulum Linnaeus, 1758

##### Notes

Types of substrate: hard bottom. Depth / bathymetric range: 0-10 m. Station code: D1(10) D2(0); D3(0, 5, 10).

#### Chthamalidae

#### Chthamalus cirratus

Darwin, 1854

##### Notes

Types of substrate: hard bottom. Depth / bathymetric range: 0-5 m. Station code: D1(0); D2(0); D3(0, 5); D4(0).

#### Notochthamalus scabrosus

(Darwin, 1854)

Chthamalus scabrosus Darwin, 1854

##### Notes

Types of substrate: hard bottom. Depth / bathymetric range: 0-10 m. Station code: D2(0, 10); D3(0); D4(0); D5(0).

### Brachiopoda

#### Discinidae

#### Discinisca lamellosa

(Broderip, 1833)

Orbicula lamellosa Broderip, 1833

##### Notes

Types of substrate: hard and soft bottoms. Depth / bathymetric range: 0-10 m. Station code: BT4N(8); D1(5, 10); D2(5, 10); D3(0, 5, 10); D4(5, 10).

### Bryozoa

#### Bugulidae

#### Bugula neritina

(Linnaeus, 1758)

Sertularia neritina Linnaeus, 1758

##### Notes

Types of substrate: soft bottom. Depth / bathymetric range: 10-15 m. Station code: BT1N(10, 12); BT3N(15). Remarks: It is a biofouling species and considered as non-native in Australia and Europe (Ryland et al. 2011).

### Cnidaria

#### Actiniidae

#### Oulactis concinnata

(Drayton in Dana, 1846)

Metridium concinnatum Drayton in Dana, 1846

##### Notes

Types of substrate: hard bottom. Depth / bathymetric range: 0-5 m. Station code: D1(5); D3(0,5).

#### Sagartiidae

#### Anthothoe chilensis

(Lesson, 1830)

Actinia chilensis Lesson, 1830

##### Notes

Types of substrate: hard and soft bottoms. Depth / bathymetric range: 0-10 m. Station code: BT1S(10); BT2N(10); BT2S(8, 10); BT4N(0); D1(0, 5, 10); D2(10); D3(0, 5, 10); D4(0); D5(0).

### Echinodermata

#### Arbaciidae

#### Arbacia spatuligera

(Valenciennes, 1846)

Echinus spatuliger Valenciennes, 1846

##### Notes

Types of substrate: hard bottom. Depth / bathymetric range: 5-10 m. Station code: D4(5, 10); D5(5).

#### Tetrapygus niger

(Molina, 1782)

Echinus niger Molina, 1782

##### Notes

Types of substrate: hard bottom. Depth / bathymetric range: 0-10 m. Station code: D1(0, 5, 10); D2(0, 5, 10); D3(0, 5, 10); D4(0, 5, 10); D5(0, 5).

#### Echinometridae

#### Caenocentrotus gibbosus

(L. Agassiz, in L. Agassiz & Desor, 1846)

Echinus (Toxopneustes) gibbosus L. Agassiz & Desor, 1846

##### Notes

Types of substrate: hard bottom. Depth / bathymetric range: 0-10 m. Station code: D1(0, 5, 10); D2(0, 5); D3(0); D5(5).

#### Parechinidae

#### Loxechinus albus

(Molina, 1782)

Echinus albus Molina, 1782

##### Notes

Types of substrate: hard bottom. Depth / bathymetric range: 5-10 m. Station code: D1(5, 10); D2(5).

#### Ophiactidae

#### Ophiactis kroeyeri

Lütken, 1856

##### Notes

Types of substrate: hard and soft bottoms. Depth / bathymetric range: 0-12 m. Station code: BT1S(10); BT2S(12); BT4N(8, 10); D1(0, 5, 10); D2(0, 5, 10); D3(0, 5, 10); D4(0, 5, 10); D5(0).

### Molusca

#### Hiatellidae

#### Hiatella arctica

(Linnaeus, 1767)

Mytilus rugosus Linnaeus, 1767

##### Notes

Types of substrate: hard bottom. Depth / bathymetric range: 10 m. Station code: D1(10); D3(10).

#### Pharidae

#### Ensis macha

(Molina, 1782)

Solen scalprum King, 1832

##### Notes

Material examined: Fig. 2. The shell is large ensiform, its valves are equal, narrow and long, parallel border and surface smoothly arched. The anterior border is rounded, while the posterior border is slightly truncated. The umbos are close to the previous border. Externally the periostracum is thin, yellowish to greenish-coffee. The hinge has three cardinal teeth, two in the left valve and one in the right valve. The pallial sinus is broad and short, located towards the posterior end. Types of substrate: soft bottom. Depth / bathymetric range: 8-15 m. Station code: BT1N(8, 10); BT1S(8); BT2N(8, 12, 15); BT2S(8, 10); BT3N(8, 10, 15); BT4N(8).

#### Pholadidae

#### Barnea subtruncata

(G. B. Sowerby I, 1834)

Pholas subtruncata G.B. Sowerby I, 1834

##### Notes

Types of substrate: hard bottom. Depth / bathymetric range: 10 m. Station code: D3(10).

#### Mytilidae

#### Aulacomya atra

(Molina, 1782)

Mytilus ater Molina, 1782

##### Notes

Types of substrate: hard bottom. Depth / bathymetric range: 5 m. Station code: D2(5).

#### Brachidontes granulatus

(Hanley, 1843)

Mytilus granulatus Hanley, 1843

##### Notes

Types of substrate: hard bottom. Depth / bathymetric range: 0-5 m. Station code: D1(0, 5); D2(0); D3(0).

#### Perumytilus purpuratus

(Lamarck, 1819)

Modiola purpurata Lamarck, 1819

##### Notes

Types of substrate: hard bottom. Depth / bathymetric range: 0-10 m. Station code: D1(0, 5); D2(0); D3(0, 5, 10); D4(0, 5); D5(0).

#### Semimytilus algosus

(Gould, 1850)

Mytilus algosus Gould, 1850

##### Notes

Types of substrate: hard and soft bottoms. Depth / bathymetric range: 0-15 m. Station code: BT1N(10, 12, 15); BT1S(8, 10, 12, 15); BT2N(8, 10, 12, 15); BT2S(10, 12, 15); BT3N(10); BT3S(0, 8, 10); BT4N(8, 15); D1(0, 5, 10); D2(0, 5, 10); D3(0, 5, 10); D4(0, 5, 10); D5(0).

#### Donacidae

#### Donax obesulus

Reeve, 1854

##### Notes

Types of substrate: soft bottom. Depth / bathymetric range: 8 m. Station code: BT1N(8); BT1S(8); BT2N(8); BT2S(8); BT3S(8).

#### Lasaeidae

#### Lasaea petitiana

(Récluz, 1843)

Poronia petitiana Récluz, 1843

##### Notes

Types of substrate: hard and soft bottoms. Depth / bathymetric range: 5-10 m. Station code: BT1N(10); BT2N(15); D2(10); D3(5, 10).

#### Mactridae

#### Mactrotoma velata

(Philippi, 1849)

Mactra velata Philippi, 1849

##### Notes

Types of substrate: soft bottom. Depth / bathymetric range: 10 m. Station code: BT3S(10).

#### Mulinia edulis

(King, 1832)

Mactra edulis King, 1832

##### Notes

Types of substrate: soft bottom. Depth / bathymetric range: 8-15 m. Station code: BT1N(10, 12, 15); BT1S(8, 10, 12, 15); BT2N(8, 10, 12, 15); BT2S(8, 10, 12, 15); BT3N (10, 12, 15); BT3S (8, 10, 15); BT4N(8, 10, 15).

#### Veneridae

#### Petricola olssoni

Bernard, 1983

##### Notes

Types of substrate: hard and soft bottoms. Depth / bathymetric range: 0-15 m. Station code: BT1N(10, 12); BT1S(8); BT3N(15); BT3S(8, 15); D1(0, 5,10); D2(5, 10); D3(0, 5, 10); D4(0, 5).

#### Fissurellidae

#### Fissurella crassa

Lamarck, 1822

##### Notes

Types of substrate: hard bottom. Depth / bathymetric range: 0-5 m. Station code: D1(0); D2(0); D3(0); D4(0, 5).

#### Fissurella latimarginata

Sowerby, 1835

##### Notes

Material examined: Fig. 2. Conical shell and sharpened in the front end, medium-sized apical foramen oval, the external surface is ornamented with thin and little spaced radial striae in a dark purple background. The shell is white from the inside, with thick, uniform and purple border. The sides of the foot and mantle are of an intense black colour, with yellow prolongations in the border of the mantle. Its tentacles are deep yellow. Types of substrate: hard bottom. Depth / bathymetric range: 0-10 m. Station code: D1(0, 5); D2(0, 10).

#### Fissurella limbata

Sowerby, 1835

##### Notes

Types of substrate: hard bottom. Depth / bathymetric range: 0-10 m. Station code: D1(0, 5); D2(0, 5); D3(0, 5, 10); D4(0); D5(0).

#### Fissurella maxima

Sowerby, 1834

##### Notes

Types of substrate: hard bottom. Depth / bathymetric range: 0-10 m. Station code: D2(0, 5); D3(0, 10); D4(0).

#### Fissurella peruviana

Lamarck, 1822

##### Notes

Types of substrate: hard bottom. Depth / bathymetric range: 0-10 m. Station code: D1(0); D2(0, 10); D3(0) D4(0).

#### Lottiidae

#### Lottia orbignyi

(Dall, 1909)

Acmaea orbignyi Dall, 1909

##### Notes

Types of substrate: hard bottom. Depth / bathymetric range: 0 m. Station code: D1(0); D4(0).

#### Scurria ceciliana

(Orbigny, 1841)

Patella ceciliana Orbigny, 1841

##### Notes

Types of substrate: hard bottom. Depth / bathymetric range: 0 m. Station code: D4(0).

#### Scurria variabilis

(Sowerby, 1839)

Lottia variabilis Sowerby, 1839

##### Notes

Types of substrate: hard bottom. Depth / bathymetric range: 0 m. Station code: D1(0); D4(0).

#### Scurria viridula

(Lamarck, 1822)

Patella viridula Lamarck, 1819

##### Notes

Types of substrate: hard bottom. Depth / bathymetric range: 0 m. Station code: D4(0).

#### Siphonariidae

#### Siphonaria lessonii

Blainville, 1827

##### Notes

Types of substrate: hard bottom. Depth / bathymetric range: 0 m. Station code: D4(0).

#### Tegulidae

#### Tegula atra

(Lesson, 1830)

Trochus ater Lesson, 1830

##### Notes

Types of substrate: hard bottom. Depth / bathymetric range: 0-10 m. Station code: D1(0, 5, 10); D2(0, 5, 10); D4(0, 5); D5(0, 5).

#### Tegula euryomphala

(Jonas, 1844)

Trochus euryomphalus Jonas, 1844

##### Notes

Types of substrate: hard bottom. Depth / bathymetric range: 5 m. Station code: D1(5); D2(5).

#### Tegula luctuosa

(Orbigny, 1841)

Trochus luctuosus Orbigny, 1841

##### Notes

Types of substrate: hard bottom. Depth / bathymetric range: 5-10 m. Station code: D1(5, 10); D2(5).

#### Tegula tridentata

(Potiez & Michaud, 1838)

Monodonta tridentata Potiez & Michaud, 1838

##### Notes

Types of substrate: hard bottom. Depth / bathymetric range: 0-5 m. Station code: D1(0); D2(5).

#### Turbinidae

#### Prisogaster niger

(W. Wood, 1828)

Turbo niger W. Wood, 1828

##### Notes

Types of substrate: hard bottom. Depth / bathymetric range: 0-10 m. Station code: D1(0, 5); D2(0, 5, 10); D3(5); D4(5); D5(0, 5).

#### Caecidae

#### Caecum chilense

Stuardo, 1962

##### Notes

Types of substrate: hard bottom. Depth / bathymetric range: 5 m. Station code: D4(5).

#### Calyptraeidae

#### Crepipatella dilatata

(Lamarck, 1822)

Crepidula dilatata Lamarck, 1822

##### Notes

Types of substrate: hard bottom. Depth / bathymetric range: 0-10 m. Station code: D1(0, 5, 10); D2(0, 5, 10); D3(0, 5, 10); D4(0, 5, 10); D5(0).

#### Trochita trochiformis

(Born, 1778)

Turbo trochiformis Born, 1778

##### Notes

Types of substrate: hard bottom. Depth / bathymetric range: 10 m. Station code: D1(10).

#### Littorinidae

#### Echinolittorina peruviana

(Lamarck, 1822)

Phasianella peruviana Lamarck, 1822

##### Notes

Types of substrate: hard bottom. Depth / bathymetric range: 0 m. Station code: D5(0).

#### Naticidae

#### Neverita didyma

(Röding, 1798)

Albula didyma Röding, 1798

##### Notes

Types of substrate: soft bottom. Depth / bathymetric range: 8-15 m. Station code: BT1N(10, 12, 15); BT1S(8, 10, 12, 15); BT2N(8, 12,15); BT2S(8, 10 12, 15); BT3N(15); BT3S(8, 10, 15); BT4N(10, 15).

#### Sinum cymba

(Menke, 1828)

Natica cymba Menke, 1828

##### Notes

Types of substrate: soft bottom. Depth / bathymetric range: 8-12 m. Station code: BT1S(8, 10, 12); BT3S(8).

#### Columbellidae

#### Alia unifasciata

(G. B. Sowerby I, 1832)

Columbella unifasciata G. B. Sowerby I, 1832

##### Notes

Types of substrate: hard bottom. Depth / bathymetric range: 5 m. Station code: D1(5).

#### Muricidae

#### Concholepas concholepas

(Bruguière, 1789)

Buccinum concholepas Bruguière, 1789

##### Notes

Types of substrate: hard bottom. Depth / bathymetric range: 0-5 m. Station code: D1(0, 5); D2(0,5).

#### Crassilabrum crassilabrum

(Sowerby, 1834)

Murex crassilabrum Sowerby, 1834

##### Notes

Types of substrate: hard bottom. Depth / bathymetric range: 5-10 m. Station code: D1(5); D2(10).

#### Stramonita haemastoma

(Linnaeus, 1767)

Buccinum haemastoma Linnaeus, 1767

##### Notes

Types of substrate: hard bottom. Depth / bathymetric range: 0-10 m. Station code: D1(0, 5, 10); D2(5, 10); D3(5, 10); D4(0); D5(0).

#### Thaisella chocolata

(Duclos, 1832)

Purpura chocolata Duclos, 1832

##### Notes

Types of substrate: hard bottom.Depth / bathymetric range: 0-10 m. Station code: D1(0); D2(5, 10); D3(0, 5, 10).

#### Xanthochorus buxeus

(Broderip, 1833)

Murex buxeus Broderip in Broderip & Sowerby, 1833

##### Notes

Types of substrate: hard bottom. Depth / bathymetric range: 5-10 m. Station code: D1(5, 10).

#### Nassaridae

#### Nassarius dentifer

(Powys, 1835)

Nassa dentifera Powys, 1835

##### Notes

Types of substrate: hard and soft bottoms. Depth / bathymetric range: 8-12 m. Station code: BT1S(10); BT2N(10), BT2S(8, 10, 12); D2(10).

#### Goniodorididae

#### Okenia luna

Millen, Schrödl, Vargas & Indacochea, 1994

##### Notes

Types of substrate: hard bottom. Depth / bathymetric range: 10 m. Station code: D2(10).

#### Dotidae

#### Doto uva

Marcus, 1955

##### Notes

Types of substrate: hard bottom. Depth / bathymetric range: 0-10 m. Station code: D2(5); D3(0, 5, 10).

#### Chaetopleuridae

#### Chaetopleura hennahi

(Gray, 1828)

Chiton hennahi Gray, 1828

##### Notes

Types of substrate: hard bottom. Depth / bathymetric range: 5 m. Station code: D4(5).

#### Chitonidae

#### Acanthopleura echinata

(Barnes, 1824)

Chiton echinatus Barnes, 1824

##### Notes

Types of substrate: hard bottom. Depth / bathymetric range: 0 m. Station code: D2(0).

#### Chiton cumingsii

Frembly, 1827

##### Notes

Types of substrate: hard bottom. Depth / bathymetric range: 0-10 m. Station code: D1(0, 5, 10); D2(0, 5, 10); D3(0, 10); D4(0).

#### Chiton granosus

Frembly, 1827

##### Notes

Types of substrate: hard bottom. Depth / bathymetric range: 0-10 m. Station code: D1(0); D2(0, 10); D3(0, 10); D4(0).

## Analysis

We recorded 162 species and subspecies of marine macroinvertebrates in the submerged soft and artificial hard substrates around the PERU LNG marine terminal and surrounding area between June 2011 and June 2015 (Suppl. material 2). In soft-substrate sampling sites, we recorded 71 species. For these sites, the accumulation curve appeared asymptotic (Suppl. material 3) and the richness estimator Chao2 estimated that 89.9% of expected species were detected in our sampling (Chao2=78.892, 89.99%). In hard-substrate sampling sites, we recorded 131 species during the five years of surveys. The accumulation curve appeared nearly asymptotic (Suppl. material 4) and the richness estimator indicated that 89.7% of expected species were detected by our sampling effort (Chao2=145.933, 89.76%).

The Polychaeta was the group with the highest number of species (61 spp.), followed by Crustacea and Mollusca with 47 and 45 species, respectively. Less numerous in species, but present in the study area, were the phyla Brachiopoda, Bryozoa, Cnidaria and Echinodermata (one to five species). The photographs for some of the species listed in this study are presented in Fig. 2.

## Discussion

This study reports the diversity of macrobenthonic species associated with the coastal soft and hard bottom habitats around PERU LNG marine terminal in central Peru. In general, species richness and taxonomic composition observed in our study area are similar to other upwelling areas, north of the terminal, like Ancon Bay (Tarazona et al. 1988, Tarazona 1990) and Chancay (L. Quipúzcoa, pers. comm.) in Peru and south of the terminal, like Independence Bay in Peru (Tarazona et al. 1996) and Coloso Bay (Carrasco 1997) and Mejillones Bay (Laudien et al. 2007) on the coast of Chile. We observed, however, a slight increase in richness in the sampling sites immediately adjacent to the marine terminal, compared to the rest of the sampling sites. This was due to the presence of infrastructure. In general, large coastal marine infrastructure like docks, piers and breakwaters, have an important role in attracting benthic fauna, just like artificial reefs do (Lincoln-Smith et al. 1994). The three-dimensionality of the structures creates different types of microhabitats likely to be colonised by species with different habitat preferences. Similar to what other studies have observed for coastal macroinvertebrate communities, species richness decreased with depth (Tarazona et al. 1996, Tarazona 1990).

We highlight the report of a new Polychaeta species, *Myrianida paredesi*, described from specimens obtained from biofouling from main pier piles at PERU LNG marine terminal (Aguirre et al. 2015). Areas with regular maritime traffic, as is the case for our study area, are likely to be colonised by non-native biofouling species given the spatial range of microhabitats that offer these artificial structures. In this study, we have been able to report eight species considered as non-native (*Caprella scaura, Elasmopus rapax, Monocorophium insidiosum, Monocorophium acherusicum, Polydora websteri, Ancinus brasiliensis, Hepatus lineatus, Bugula neritina*). The most likely vector of introduction may be the maritime traffic occurring along the coast of Peru.

We detected species in our study area (but not listed here) that were challenging to identify because their presence was limited to individuals at early developmental stages (i.e. juveniles), they were present in low numbers or because of taxonomic complexity. These putative species include *Abarenicola affinis chilensis*, *Capitella capitata*, *Cirratulus megalus*, *Dodecaceria opulens*, *Eunice pelamidis*, *Hemipodia simplex*, *Kinbergonuphis microcephala*, *K. multidentata*, *Lumbrineris annulata*, *Magelona phyllisae*, *Paleanotus chrysolepis*, *Phymactis clematis*, *Pisione oerstedii*, *Polydora pygidialis*, *Scoletoma tetraura*, *Syllis gracilis*, *Thoracophelia mucronata* and *Spiophanes norrisi*. We recommend an increased sampling effort as well as an extensive review to confirm their presence in the area. Further, the application of molecular tools (i.e. barcode sequencing) could be integrated into the analyses to help improving biodiversity assessments (e.g. Rosas et al. 2018) and for resolving taxonomic conflicts (e.g. Hebert and Gregory 2005). Molecular tools offer additional benefits like the effective detection of non-indigenous species (e.g. Zaiko et al. 2015) and improving assessment of the health of marine ecosystems (Sigamani et al. 2016). Considering the high complexity, variability and productivity of the Peruvian coastal upwelling system, this study helps to increase the understanding of the local marine biodiversity and serves as a baseline for monitoring of the spatial and temporal changes in the diversity and composition of coastal macrobenthic communities.

## Supplementary Material

Supplementary material 1 Table 1. Sampling sites at central coast of Peru and influence area of PERU LNG marine terminal, including depth(s), type of substrate and geographic decimal coordinates. Data type: Formatted text
Brief description: Word table with geographic information of sampling sites
File: oo_221022.docx V. Tasso, M. El Haddad, C. Assadi, R. Canales, L. Aguirre, X. Velez-Zuazo

Supplementary material 2 Macrobenthos species ocurrence list Data type: Darwin Core Archive (.zip) of occurrence data and associated metadata
Brief description: Resource link: http://ipt.pensoft.net/resource?r=macrobenthos_bmapperu&v=1.2
File: oo_225921.zip Tasso V, El Haddad M, Assadi C, Canales R, Aguirre L, Velez-Zuazo X

Supplementary material 3 Species accumulation curve using rarefaction method for macrobenthos reported at soft-bottom sampling sites. Light blue shaded area indicates 95% confidence interval. Data type: image
File: oo_226161.jpeg Tasso V, El Haddad M, Assadi C, Canales R, Aguirre L, Velez-Zuazo X

Supplementary material 4 Species accumulation curve using rarefaction method for macrobenthos reported at hard-bottom sampling sites. Light blue shaded area indicates 95% confidence interval. Data type: imape
File: oo_226162.jpeg Tasso V, El Haddad M, Assadi C, Canales R, Aguirre L, Velez-Zuazo X

## Figures and Tables

**Figure 1a.**
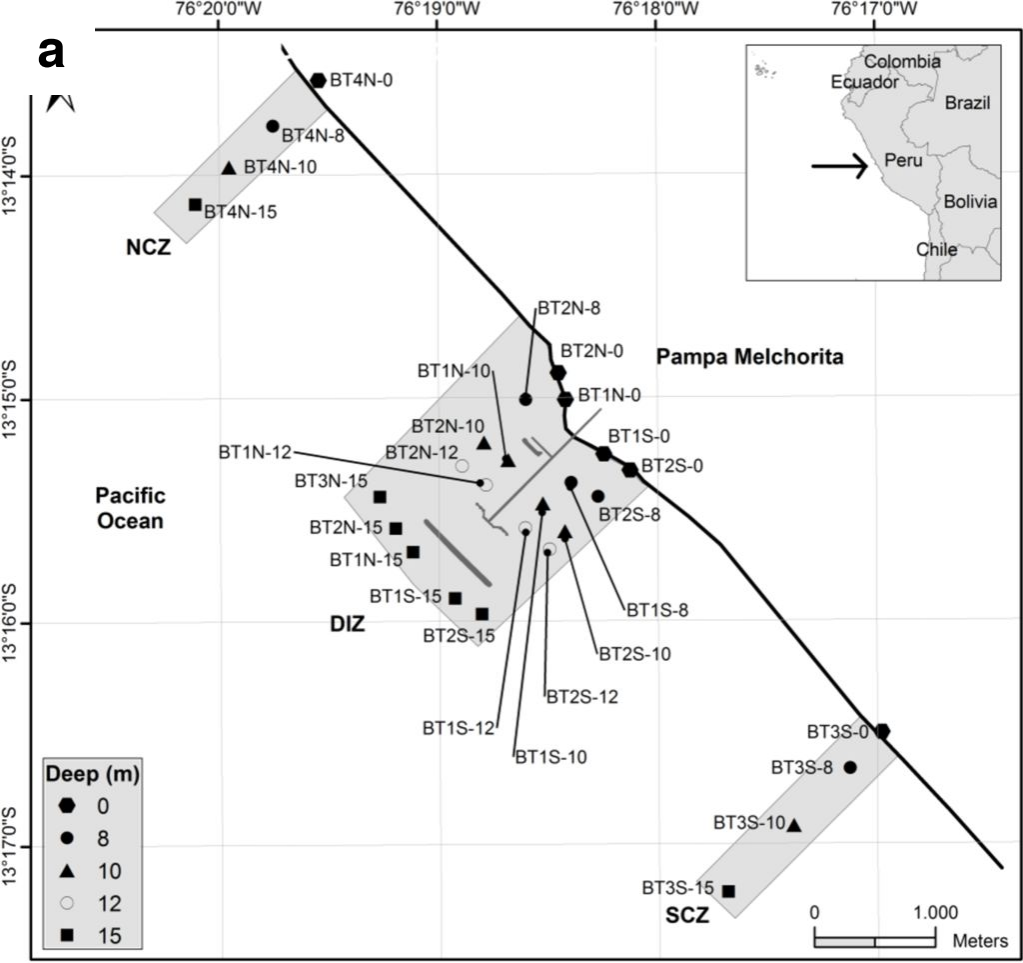
Soft bottom sites.

**Figure 1b.**
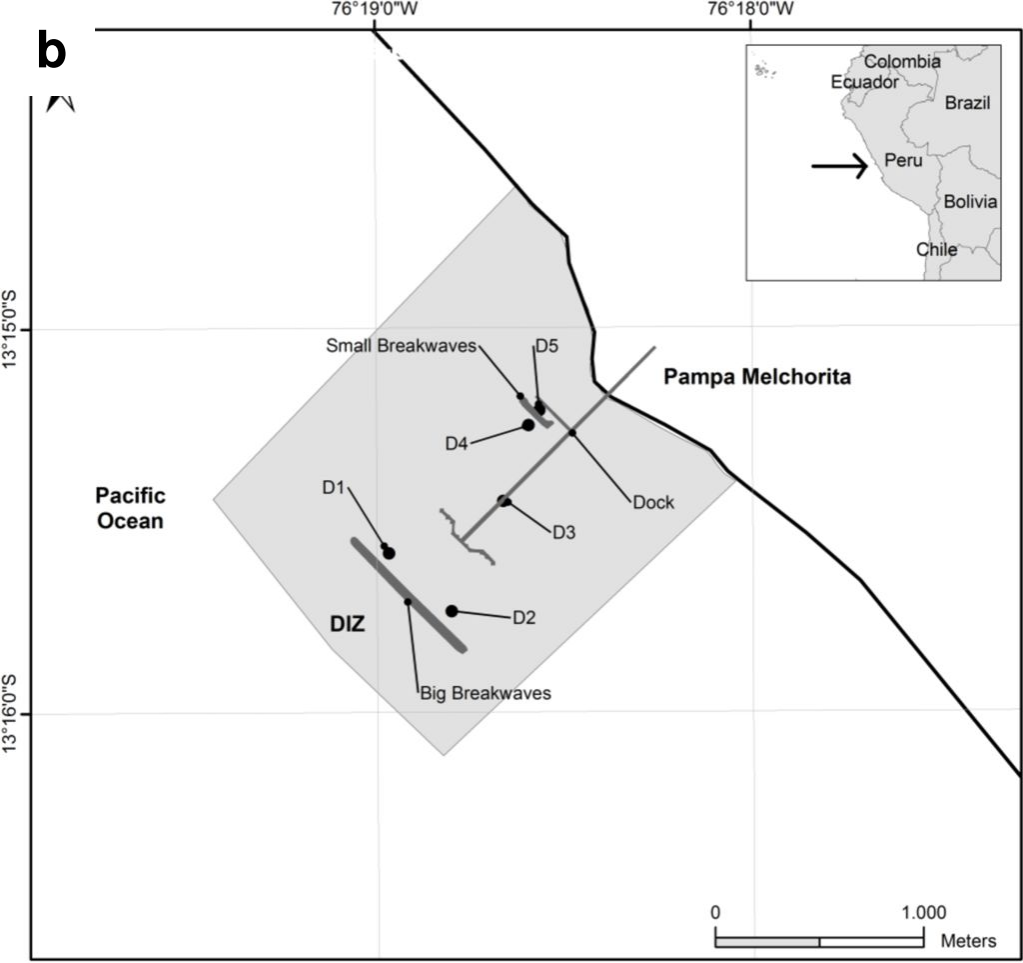
Hard bottom sites.
