# Supplementary material for: Macrobenthic fauna from an upwelling coastal area of Peru (Warm Temperate South-eastern Pacific province -Humboldtian ecoregion)
Source: Biodivers Data J. 2018 Sep 10;(6):e28937. doi: 10.3897/BDJ.6.e28937 (PMC6160799; doi:10.3897/BDJ.6.e28937)
Supplement: Supplementary material 1 — Table 1. Sampling sites at central coast of Peru and influence area of PERU LNG marine terminal, including depth(s), type of substrate and geographic decimal coordinates. [file bdj-06-e28937-s001.docx]

Table 1. Sampling sites at central coast of Peru and influence area of PERU LNG marine terminal, including depth(s), type of substrate, and geographic decimal coordinates.

| Site | Depth (m) | Substrate | Latitude S | Longitude W |
| --- | --- | --- | --- | --- |
| D1 | 0, 5, 10 | Hard bottom | 13° 15,58585' | 76° 18,96651' |
| D2 | 0, 5, 10 | Hard bottom | 13° 15,73695' | 76° 18,80064' |
| D3 | 0, 5, 10 | Hard bottom | 13° 15,35040' | 76° 18,55160' |
| D4 | 0, 5, 10 | Hard bottom | 13° 15,24060' | 76° 18,59587' |
| D5 | 0, 5 | Hard bottom | 13° 15,21473' | 76° 18,56472' |
| BT4N | 0 | Soft bottom | 13° 13,58566' | 76° 19,54431' |
| BT2N | 0 | Soft bottom | 13° 14,88225' | 76° 18,45275' |
| BT1N | 0 | Soft bottom | 13° 15,01582' | 76° 18,42633' |
| BT1S | 0 | Soft bottom | 13° 15,25111' | 76° 18,23987' |
| BT2S | 0 | Soft bottom | 13° 15,32548' | 76° 18,12177' |
| BT3S | 0 | Soft bottom | 13° 16,50187' | 76° 16,97173' |
| BT4N | 8 | Soft bottom | 13° 13,77985' | 76° 19,75133' |
| BT2N | 8 | Soft bottom | 13° 15,00625' | 76° 18,60070' |
| BT1N | 8 | Soft bottom | 13° 15,11250' | 76° 18,50825' |
| BT1S | 8 | Soft bottom | 13° 15,38049' | 76° 18,39506' |
| BT2S | 8 | Soft bottom | 13° 15,44405' | 76° 18,27191' |
| BT3S | 8 | Soft bottom | 13° 16,66274' | 76° 17,12432' |
| BT4N | 10 | Soft bottom | 13° 13,98719' | 76° 19,93406' |
| BT2N | 10 | Soft bottom | 13° 15,22441' | 76° 18,79125' |
| BT1N | 10 | Soft bottom | 13° 15,28573' | 76° 18,68138' |
| BT1S | 10 | Soft bottom | 13° 15,47266' | 76° 18,51017' |
| BT2S | 10 | Soft bottom | 13° 15,58660' | 76° 18,39892' |
| BT3S | 10 | Soft bottom | 13° 16,93643' | 76° 17,38549' |
| BT2N | 12 | Soft bottom | 13° 15,32592' | 76° 18,88094' |
| BT1N | 12 | Soft bottom | 13° 15,37844' | 76° 18,79429' |
| BT1S | 12 | Soft bottom | 13° 15,58454' | 76° 18,58774' |
| BT2S | 12 | Soft bottom | 13° 15,67670' | 76° 18,49244' |
| BT4N | 15 | Soft bottom | 13° 14,10827' | 76° 20,11854' |
| BT3N | 15 | Soft bottom | 13° 15,45354' | 76° 19,27202' |
| BT2N | 15 | Soft bottom | 13° 15,60462' | 76° 19,21801' |
| BT1N | 15 | Soft bottom | 13° 15,69207' | 76° 19,08946' |
| BT1S | 15 | Soft bottom | 13° 15,91858' | 76° 18,92178' |
| BT2S | 15 | Soft bottom | 13° 15,98923' | 76° 18,79092' |
| BT3S | 15 | Soft bottom | 13° 17,19530' | 76° 17,68148' |
| BT1N | 0 | Soft bottom | 13° 15,56908' | 76° 18,42929' |
| BT2N | 0 | Soft bottom | 13° 14,88225' | 76° 18,45275' |
| BT1S | 0 | Soft bottom | 13° 15,25111' | 76° 18,23987' |
| BT2S | 0 | Soft bottom | 13° 15,32548' | 76° 18,12177' |
| BT3S | 0 | Soft bottom | 13° 16,50187' | 76° 16,97173' |
| BT4N | 0 | Soft bottom | 13° 13,58566' | 76° 19,54431' |
| MU | 17 | Soft bottom | 13° 15,56726' | 76° 18,78478' |
