# Supplementary figures and images for: Macrobenthic fauna from an upwelling coastal area of Peru (Warm Temperate South-eastern Pacific province -Humboldtian ecoregion)
Source: Biodivers Data J. 2018 Sep 10;(6):e28937. doi: 10.3897/BDJ.6.e28937 (PMC6160799; doi:10.3897/BDJ.6.e28937)

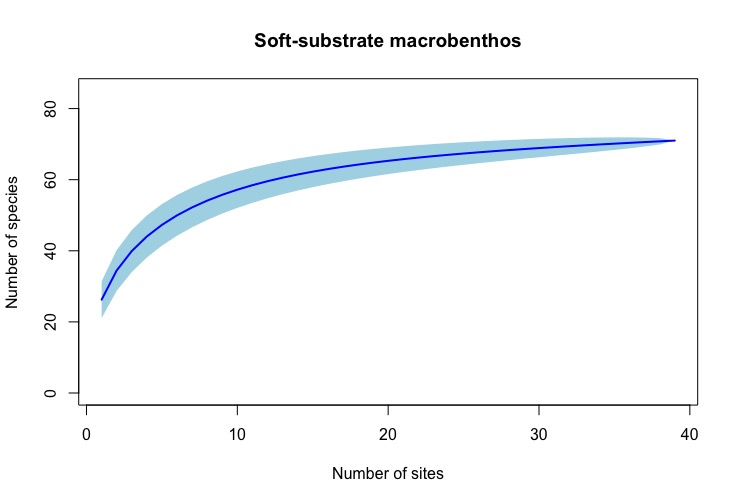

Supplement: Supplementary material 3 — Species accumulation curve using rarefaction method for macrobenthos reported at soft-bottom sampling sites. Light blue shaded area indicates 95% confidence interval. [file bdj-06-e28937-s003.jpeg]

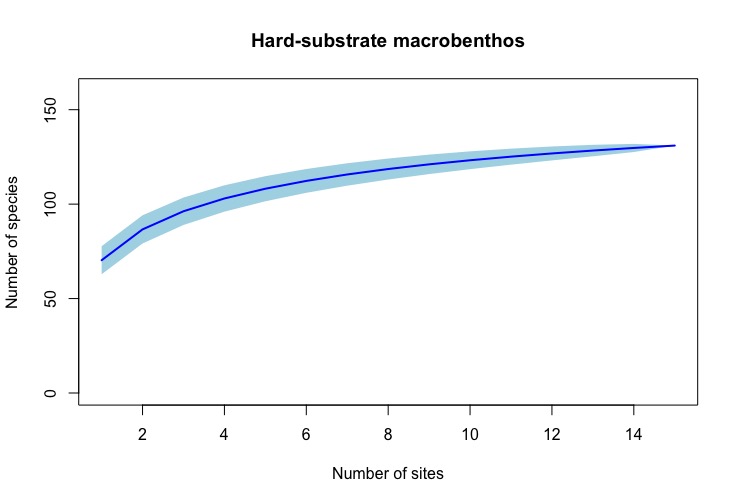

Supplement: Supplementary material 4 — Species accumulation curve using rarefaction method for macrobenthos reported at hard-bottom sampling sites. Light blue shaded area indicates 95% confidence interval. [file bdj-06-e28937-s004.jpeg]
